# The impact of cardiopulmonary hemodynamic factors in volumetry for pulmonary nodule management

**DOI:** 10.1186/s12880-022-00774-w

**Published:** 2022-03-18

**Authors:** Erique Guedes Pinto, Diana Penha, Bruno Hochhegger, Colin Monaghan, Edson Marchiori, Luís Taborda-Barata, Klaus Irion

**Affiliations:** 1 Universidade da Beira Interior, Covilhã, Portugal; 2 Imaging Department, Liverpool Heart and Chest Hospital NHS Foundation Trust: Liverpool, Liverpool, UK; 3 Pontifical Catholic University of Rio Grande Do Sul, Porto Alegre, Brazil; 4 Federal University of Rio de Janeiro, Rio de Janeiro, Brazil; 5 Imaging Department, University of Manchester, Manchester, UK

**Keywords:** Cardiac-gated imaging techniques [E01.370.350.130.500, Lung neoplasms [C04.588.894.797.520, Pulmonary circulation [G09.330.100.770, Radiographic image interpretation, computer-assisted [E01.158.600.680

## Abstract

**Background:**

The acceptance of coronary CT angiogram (CCTA) scans in the management of stable angina has led to an exponential increase in studies performed and reported incidental findings, including pulmonary nodules (PN). Using low-dose CT scans, volumetry tools are used in growth assessment and risk stratification of PN between 5 and 8 mm in diameter. Volumetry of PN could also benefit from the increased temporal resolution of CCTA scans, potentially expediting clinical decisions when an incidental PN is first detected on a CCTA scan, and allow for better resource management and planning in a Radiology department. This study aims to investigate how cardiopulmonary hemodynamic factors impact the volumetry of PN using CCTA scans. These factors include the cardiac phase, vascular distance from the main pulmonary artery (MPA) to the nodule, difference of the MPA diameter between systole and diastole, nodule location, and cardiomegaly presence.

**Materials and methods:**

Two readers reviewed all CCTA scans performed from 2016 to 2019 in a tertiary hospital and detected PN measuring between 5 and 8 mm in diameter. Each observer measured each nodule using two different software packages and in systole and diastole. A multiple linear regression model was applied, and inter-observer and inter-software agreement were assessed using intraclass correlation.

**Results:**

A total of 195 nodules from 107 patients were included in this retrospective, cross-sectional and observational study. The regression model identified the vascular distance (p < 0.001), the difference of the MPA diameter between systole and diastole (p < 0.001), and the location within the lower or posterior thirds of the field of view (p < 0.001 each) as affecting the volume measurement. The cardiac phase was not significant in the model. There was a very high inter-observer agreement but no reasonable inter-software agreement between measurements.

**Conclusions:**

PN volumetry using CCTA scans seems to be sensitive to cardiopulmonary hemodynamic changes independently of the cardiac phase. These might also be relevant to non-gated scans, such as during PN follow-up. The cardiopulmonary hemodynamic changes are a new limiting factor to PN volumetry. In addition, when a patient experiences an acute or deteriorating cardiopulmonary disease during PN follow-up, these hemodynamic changes could affect the PN growth estimation.

## Keypoints

1. Cardiopulmonary hemodynamic factors affect volumetry for pulmonary nodule management.
2. The impact of hemodynamic changes in volumetry measurements is not related to the cardiac cycle phase, making them relevant even in non-gated scans.
3. During follow-up of a pulmonary nodule, hemodynamic changes (such as decompensated heart failure) could potentially impact growth estimation.

## Background

The introduction of coronary CT angiograms (CCTA) in the diagnosis and management of stable coronary artery disease (CAD) has improved clinical outcomes and reduced the need for invasive coronary angiography [1]. As a result, CCTA is now established as a first-line examination for stable CAD [1, 2]. This has exponentially increased the number of CCTA examinations performed and the associated incidental findings [3].

Pulmonary nodules (PN) are among the most common incidental extracardiac findings on CCTA (up to 28% of scans) and require follow-up, even if only rarely malignant [3–5].

Several scientific societies have published guidelines and recommendations for PN management [6]. Small solid PNs are considered benign and do not require follow-up (e.g., smaller than 5 mm in diameter according to the British Thoracic Society (BTS) [7, 8] or 6 mm according to the Fleischner Society [9], the International Early Lung Cancer Action Program (I-ELCAP) [10], and the American College of Radiology (ACR, Lung-Rads) [11]. An exception is the National Comprehensive Cancer Network (NCCN), which recommends that nodules < 5 mm be followed up until the patient is no longer a candidate for definitive treatment [12]. Larger PN have a higher risk of malignancy, as evidenced by the Dutch-Belgian Lung Cancer Screening (NELSON) trial and I-ELCAP [10, 13].

In solid non-calcified PN between 5 and 8 mm in size, the growth rate is a better discriminator between benign and malignant pathology than size or morphological features [10]. The time required for a PN to double in volume at its specific growth rate is called the volume doubling time (VDT). Follow-up studies of PNs using low-dose CT scans (LD-CT) show that VDT is usually shorter than 30 days (e.g., inflammatory changes) or longer than 400 days (e.g., hamartomas) for benign pathology. In contrast, VDT tends to be between 30 and 400 days for malignant pathology [10, 14]. The further apart the two measurements are, the more reliable the growth estimate is. This is especially true for slow-growing lesions (VDT of 400 days), where intrinsic variability in the measurement may overshadow the actual growth of the lesion. However, even fast-growing (VDT of 180 days) 6 mm PN may have overlapping volume measurements with stable 6 mm PN if the follow-up period is less than three months [10].

Choosing the right time for follow-up is important. The Fleischner Society recommends waiting 6–12 months if there is a solid solitary PN with a diameter of 6–8 mm (100–250 mm^3^), or 3–6 months if there are multiple solid nodules with a diameter greater the 6 mm (> 100 mm^3^) [9].

PN follow-up is done using LD-CT scans. Suppose the PN is incidentally detected on a chest CT examination with a different protocol (e.g., CCTA). In that case, an LD-CT study should be requested as soon as possible and used as the baseline [15]. This raises the possibility of delaying the final diagnosis and justifies the use of institutional alert systems for actionable findings (such as PN), which are neither universal nor foolproof [16]. Using the initial CCTA scan as the baseline for growth assessment would allow better planning and resource management in a radiology department and reduce patient radiation exposure. Compared with LD-CT scans, the increased temporal resolution of CCTA could also improve the robustness of the measurement to cardiac motion and breathing artifacts, which are known to affect PN volumetry through available software tools [17].

All major scientific societies currently recommend automatic or semiautomatic volumetry tools for PN growth estimation, despite known limiting factors related to the nodule, adjacent structures, scanning protocol, or equipment [17]. Boll et al. published the only other study of PN volumetry in ECG-gated CT scans and suggested that cardiovascular motion, as resulting from the interaction of the cardiac cycle phase, location, and mean size of a PN, may influence PN volumetry [18].

## Methods

This study aims to investigate how factors known to be related to the cardiopulmonary circulation, namely the cardiac cycle phase during image acquisition, the distance between the MPA and the PN, change in diameter of the MPA between systole and diastole, location of the PN (concerning hydrostatic pressure and vascular cross-sectional area), and presence of cardiomegaly, affect the results of volumetry tools currently in clinical use so that we can better understand their potential applications and limitations.

The Institutional Research Committee Review Board approved this retrospective study (cross-sectional, observational, analytical) and waived the requirement for written informed consent because of the exclusive use of existing data.

### Study sample

The study sample included all consecutive CCTA examinations performed at a tertiary cardiothoracic center from 2016 to 2019. All scans were performed with the same equipment (Somatom Definition Flash; Siemens). The imaging protocol used for the CCTA examinations is shown in Table 1.

**Table 1:** Imaging protocol for coronary CT angiogram (CCTA)

CCTA Imaging protocol parameters	
Range	From the carina to the apex of the heart
Respiratory phase	Inspiration, breath-hold
Contrast enhancement	75–95 ml of Niopam 370 (iopamidol), at 5–7 ml/s
Image reconstruction	2 mm thickness, 0.75 mm overlap
Kernels	B20f smooth/mediastinum, B60f sharp/lung
Acquisition parameters	Peak kilovoltage (kVp) between 100 and 120 kV; current modulation (CareDose 4D) with 320 mAs as reference
Average acquisition time	1–2 s

The inclusion criteria comprise scans showing at least one solid non-calcified PN with a long-axis diameter between 5 and 8 mm. Exclusion criteria include the absence of an adequate systolic or diastolic phase archived in the hospital’s picture archiving and communication system (PACS), defined as 30–40% for systole and 70–80% for diastole; or if the PN was not shown in the field of view (FOV) of both systole and diastole.

### Readers and measurements

**Fig. 1:**
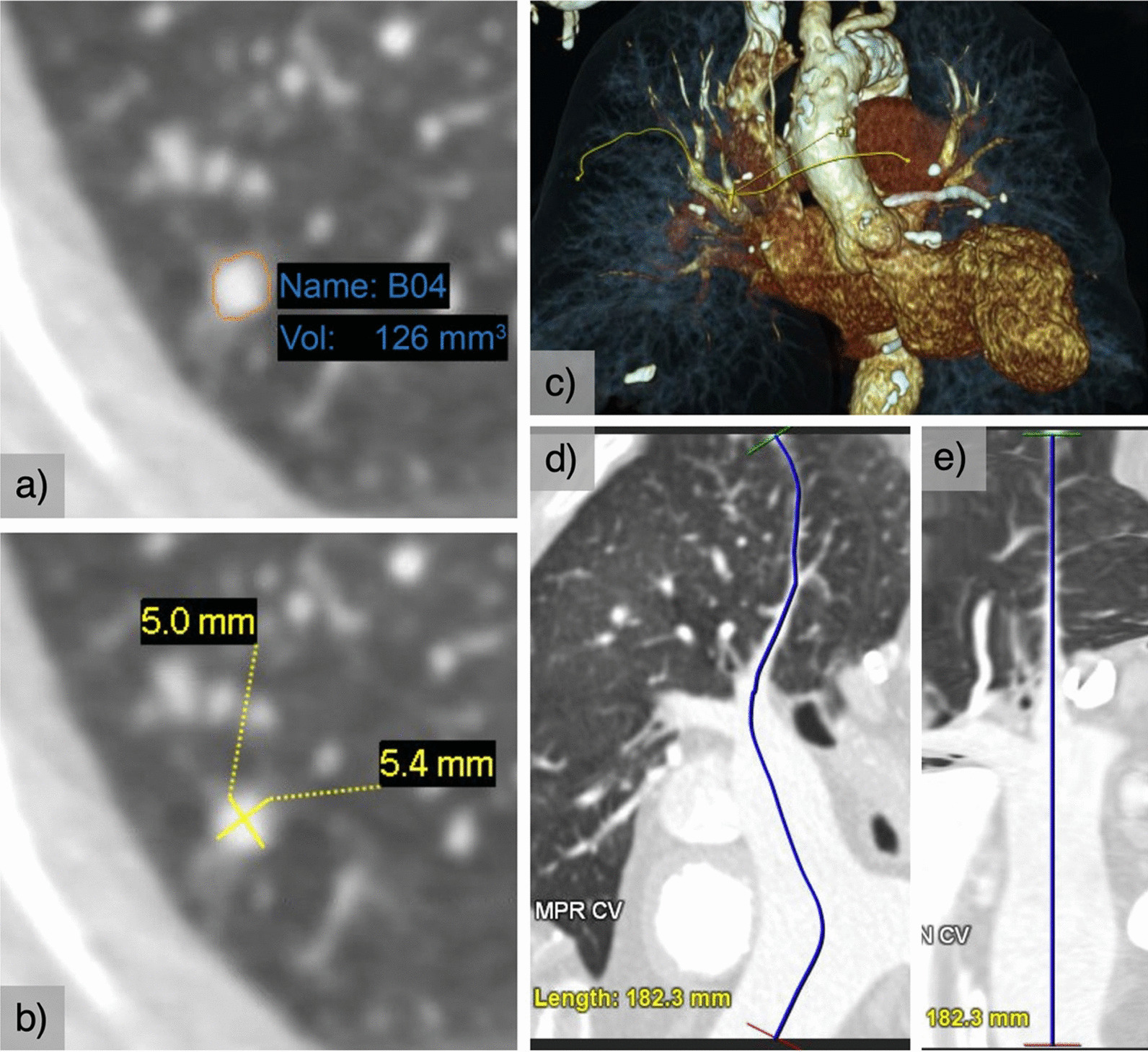
**a** Volume measurement using the volumetry tool; **b** manual long- and short-diameter measurement using electronic calipers; **c**–**e** tracking the vascular distance between the proximal MPA (at the level of the pulmonary valve) to the nodule

Two cardiothoracic radiologists with 10 (reader 1) and 5 (reader 2) years of experience identified and measured solid non-calcified PNs according to the protocol described in Fig. 1 and using the Carestream Vue PACS v 11.4.01.1011 (Carestream Health, Inc, Rochester, NY; tool 1) and Syngo via VB20 (Siemens Healthineers AG, Erlangen, Germany; tool 2) commercially available volumetric software packages based on region-growing algorithms. Both these tools performed semiautomatic segmentation by placing one seed point in the center of the nodule. The readers did not correct the resulting segmentation.

If the two readers disagreed on whether an appearance was a true nodule, a consensus decision was made between the two readers and another cardiothoracic radiologist with more than 25 years of experience.

For each nodule identified, the following information was recorded: reader; software package used; patient age and sex; failed segmentation of the nodule, defined as three consecutive failed attempts; nodule segmentation appropriateness, defined as complete PN inclusion with the exclusion of adjacent structures; semiautomatic volume measurement; long- and short-axis diameters in the axial plane, measured manually and semiautomatically, and rounded to one decimal place; distance from the MPA, at the level of the pulmonary valve, to the nodule (vascular distance) measured using curved plane reconstruction (Fig. 1); location of the PN in the axial (anterior; middle; posterior) and coronal plane (superior; middle; inferior); the diameter of the MPA in systole and diastole, measured at the level of the pulmonary valve and in the axial plane; and the presence of cardiomegaly.

### Statistical analysis

Data were analyzed using SPSS software (ver. 26.0; IBM Corporation, Armonk, NY, USA).

The inter-quartile range (IQR) method detected outliers in volume measurements. Outliers and cases where segmentation failed were excluded from further analysis.

The average of long- and short-axis manually measured diameters (average diameter) was calculated for all included cases according to the recommendations of the Fleischner Society [9]. In addition, a continuous variable representing the difference of the MPA diameter between systole and diastole measured in the axial plane and at the level of the pulmonary valve was calculated for all nodules.

A multiple linear regression model using a stepwise automatic selection of significant variables was used to predict volume measures based on the reader, software package, cardiac cycle phase, appropriateness of segmentation, average diameter, vascular distance, difference of the MPA diameter between systole and diastole, location of the PN in both axial and coronal planes, presence of cardiomegaly, age, and sex.

The intraclass correlation coefficient (ICC) with an absolute agreement-type two-way mixed model was used to assess inter-observer and inter-software agreement.

## Results

Of a total of 5973 CCTA scans performed, 4478 scans were excluded because either a systolic or diastolic phase was missing from PACS. In addition, 1357 scans were excluded because there were no qualifying solid PN in both systolic and diastolic FOV, and 31 scans were excluded after a consensus decision because they did not represent true nodules. Of the remaining 107 CCTA scans, a total of 195 solid, non-calcified nodules were identified with a long-axis diameter between 5 and 8 mm.

The mean age of the patients was 67.8 years, and the male to female ratio was 1.38 (Table 2).

**Table 2:** Patient demographics and indications for CCTA

Patient characteristics	(N = 107)
Age (years): M ± SD	67.8 ± 11.7
*Sex: n (%)*	
Male	62 (57.9%)
Female	45 (42.1%)
Male to Female ratio	1.38
*Indication for the CCTA examination: n (%)*	
Acute or chronic chest pain	47 (43.9%)
Hypertension	16 (14.9%)
Diabetes Mellitus	13 (12.1%)
Abnormal or equivocal stress test	9 (8.4%)
Dyspnea	8 (7.4%)
Pre-operative	5 (4.7%)
Abnormal ECG	3 (2.8%)
Congestive heart failure	3 (2.8%)
Palpitations	3 (2.8%)

*M* mean, *SD* standard deviation

Each PN was measured by each reader for systole and diastole and, using each software tool, resulting in a total of 1560 measurements (8 measurements per nodule).

Nodule segmentation failed in 11 measurements (1.41%) using the Carestream Vue PACS software package and 15 measurements (1.92%) using the Syngo via software package. However, the segmentation was considered appropriate more often using the Syngo via software package (n = 685; 87.8%) than the Carestream Vue PACS software package (n = 623; 79.9%).

A total of 204 measurements were identified as outliers using the IQR method and removed from further analysis. The outliers' mean volume and standard deviation were 586.8 ± 1500.8 mm^3^, with a minimum of 159 mm^3^ and a maximum of 13,300 mm^3^.

Nodules were more frequently identified in the upper third (49.2%) in the axial plane, and between the middle (39.5%) and posterior (40.5%) thirds of the FOV, in the coronal plane.

Descriptive statistical analysis is provided in Table 3.

**Table 3:** Descriptive statistics of quantitative variables

N = 1322	M ± SD	Min	Q1	Q2	Q3	Max
Volume (mm^3^)	43.527 ± 30.065	2.0	22.0	34.1	58.1	152.0
Automatic long-axis diameter (mm)	6.375 ± 1.697	3.9	5.2	6	7.2	13.0
Automatic short-axis diameter (mm)	5.386 ± 1.104	2.9	4.7	5.3	6	7.7
Manual long-axis diameter (mm)	6.101 ± 1.001	4.3	5.2	6.5	7.1	8.5
Manual short-axis diameter (mm)	4.255 ± 0.789	2.9	3.9	4.2	4.7	6.5
Average diameter (mm)	5.178 ± 0.773	3.5	4.4	5.5	5.9	7.5
Vascular distance (cm)	18.405 ± 3.178	10.6	16.0	18.4	20.7	26.4
Difference of the MPA diameter between systole and diastole (mm)	2.295 ± 1.522	0.1	1.0	2.0	3.3	6.3

MPA main pulmonary artery

The results of the regression model are presented in Table 4. The model explains 55.3% of the variation in volume measurement (R^2^ = 0.553) and yields significant results (F_8,1313_ = 202.785, p < 0.001), without significant autocorrelation (DW = 1.871) or multicollinearity (VIF < 2).

**Table 4:** Parameter estimates for the prediction of Volume

Variable	B	95% CI	t	p	Effect size
(Constant)	− 37.40	[− 48.82, − 25.98)	− 6.426	*** < 0.001	0.030
Appropriate segmentation	− 32.69	[− 37.17, − 28.20]	− 14.291	*** < 0.001	0.135
Average diameter	24.10	[22.66, 25.54]	32.824	*** < 0.001	0.451
Vascular distance	− 0.98	[− 1.42, − 0.54]	− 4.368	*** < 0.001	0.014
Difference of the MPA diameter between systole and diastole	2.26	[1.52, 2.99]	6.019	*** < 0.001	0.027
Lower third (coronal)	13.38	[9.84, 16.93]	7.410	*** < 0.001	0.040
Posterior third (axial)	7.87	[5.46, 10.29]	6.407	*** < 0.001	0.030
Sex (male as reference)	− 3.45	[− 5.79, − 1.11]	− 2.895	**0.004	0.006
Cardiomegaly	7.05	[2.59, 11.51]	3.103	**0.002	0.007
*Excluded variables*					
Cardiac cycle phase				0.348	
Reader				0.590	
Software package for Volumetry				0.341	
Middle third (axial)				0.978	
Middle third (coronal)				0.505	
Age				0.165	

*MPA* main pulmonary artery, *B* parameter coefficient, *CI* confidence interval, *t* t test

^***^p < 0.001; **p < 0.01; Effect size (partial eta square—ζ^2^)

The results show that the volume measurement increases with the average diameter (B = 24.10; CI_95_ [22.66, 25.54], p < 0.001); with the increasing difference of the MPA diameter between systole and diastole (B = 2.26; CI_95_ [1.52, 2.99], p < 0.001); when it is in the lower third (B = 13.03; CI_95_ [9.00, 17.06], p < 0.001) compared to the upper (reference) in the coronal plane; and when it is in the posterior third (B = 8.94; CI_95_ [5.64, 12.24], p < 0.001) compared to the anterior (reference) in the axial plane.

Volume measurement decreases when segmentation is considered appropriate (B =  − 32.69; CI_95_ [− 48.82, − 25.98], p < 0.001), and with increasing vascular distance (B =  − 0.98: CI_95_ [− 1.42, − 0.54], p < 0.001).

The effect size is larger for the average diameter (ζ^2^ = 0.451, large), followed by the appropriateness of segmentation (ζ^2^ = 0.135, intermediate to large), the location of the PN in the lower third (ζ^2^ = 0.040, small to intermediate) or the posterior third (ζ^2^ = 0.030, small) of the FOV, the difference of the MPA diameter between systole and diastole (ζ^2^ = 0.027, small) and for the distance between the MPA and the PN (ζ^2^ = 0.014, small).

The results also show a tendency for the nodule to be slightly larger in the presence of cardiomegaly (B = 7.05; CI_95_ [2.59, 11.51], p = 0.002) and somewhat smaller in women (B =  − 3.45; CI_95_ [− 5.79, − 1.11], p = 0.004), but with a negligible effect size.

The cardiac cycle phase is not statistically significant in our regression model (p = 0.348), and there was no statistically significant difference in volume measurements based on cardiac phase using multivariate tests (F_7,1518_ = 0.428, p = 0.885, Wilks’ lambda (Λ) = 0.998, partial η^2^ = 0.002).

Intraclass correlation analysis (Table 5) shows very high reliability between the two readers (ICC = 0.870; CI_95_ [0.850, 0.887]) with no statistically significant difference between them (F_1,764_ = 0.561, p = 0.561). There is no reasonable reliability between the two software packages (ICC = 0.059; CI_95_ [− 0.083, 0.183], p = 0.002), and there is a statistically significant difference between the measurement made with the two software tools (F_1,760_ = 9.473, p = 0.002).

**Table 5:** ICC between readers and software tools, for Volume measurement

	ICC	(95% CI)	F	p
*Between readers*				
Global Sample	0.870	[0.850, 0.887]	0.561	0.454
Systole 30–40%	0.865	[0.834, 0.889]	0.682	0.409
Diastole 70–80%	0.965	[0.958, 0.972]	0.373	0.542
*Between software packages*				
Global sample	0.059	[− 0.083, 0.183]	9.473	**0.002
Systole 30–40%	0.027	[− 0.186, 0.203]	6.302	*0.012
Diastole 70–80%	0.545	[0.441, 0.630]	12.559	*** < 0.001

*ICC* intra class coefficient, *CI* confidence interval

^***^p < 0.001; **p < 0.01; *p < 0.1

## Discussion

The true volume of in vivo pulmonary nodules is unknown, and even when those nodules are surgically excised, their volume changes because of the sudden stop in blood flow [17]. Therefore, it is our expectation that the blood flow inside and surrounding a given pulmonary nodule will be included in the segmentation result provided by the volumetry tool. In that sense, it is expected that an increase in the blood pressure felt inside the nodule will translate into an increase in the blood volume included in the segmentation.

Our study identified factors related to cardiopulmonary circulation, namely the location of the PN, the difference of the MPA diameter between systole and diastole, and vascular distance between the MPA and the PN, which were statistically associated with the measurements of volumetry tools.

These results could be explained by the propagation of vascular pressure from the heart to the nodule through the complex arterial and capillary network. Assuming the pressure difference at the MPA between systole and diastole, one might expect a similar pressure difference at the capillaries surrounding the PN and that the cardiac cycle phase during acquisition would also be important. However, our results do not support this intuition nor corroborate earlier results from Boll et al. [18]. This is only the second study investigating PN volumetry using ECG-gated scans to the best of our knowledge. The significant dependence of volume measurement on the cardiac cycle phase, reported previously, has not yet been independently validated. Differences between our study and the former include larger sample size, number of detector rows of the CT scanner (128 vs. 16 slices), and current and updated segmentation algorithms in clinical use. The independence of volume measurement from the cardiac cycle phase means that our data cannot be used to recommend any particular cardiac phase for PN volumetry on CCTA scans. It could also suggest that these hemodynamic effects could be seen in non-gated scans (such as LD-CT). The high temporal resolution of CCTA scans accounts for their sensibility to momentary cardiopulmonary hemodynamic changes (subtle differences between two phases in a single cardiac cycle). These momentary changes could be used to model clinically significant hemodynamic changes that a patient may experience when suffering from an acute or progressing cardiopulmonary disease and could be extreme enough to affect the nodule’s growth estimation. The overlap of demographic characteristics and risk factors in patients evaluated for CAD or PN follow-up makes this area of research clinically relevant.

A physiological model of cardiopulmonary hemodynamics should consider that the total time required for the pressure wave to reach the PN (transit time) depends on the complex interaction between vascular and hydrostatic pressures, vascular resistance, the distance the blood travels between leaving the right ventricle and reaching the nodule (vascular distance), and the cross-sectional area of the vessels along the way. All these factors may affect the transit time for every PN differently so that the pressure peak reaches each PN at a different cardiac cycle phase.

The dynamic change of the intravascular pressure felt around or inside the PN could change the blood volume included in the segmentation result and consequently the volume measurement. If the change to the segmentation volume is not consistent between follow-up scans, the VDT will be either under- or overestimated.

During PN follow-up, events such as the onset of pleural effusion related to congestive heart failure could alter the PN position, vascular distance (as the aerated lung parenchyma is displaced by the volume of pleural effusion), and intravascular pressure, potentially impacting subsequent volume measurements.

Chronic thromboembolism and vasculitis are associated with increased resistance and tortuosity of the distal pulmonary vessels, affecting the propagation of the intra-vascular pressure wave, and the pressure magnitude. Clinically, these hemodynamic changes can lead to pulmonary hypertension and ultimately right ventricular dysfunction. This phenomenon increases the diameter of the MPA in both systole and diastole, but predominantly in the later, as can be seen on CT, reducing the diameter difference between both cardiac phases. In contrast, pulmonary valvular regurgitation would increase this difference by predominantly reducing the diastolic pressure [19].

Changes in hydrostatic pressure (anteroposterior gradient in a patient in decubitus position on the CT scanner), changes in the vessels' cross-sectional luminal area (craniocaudal gradient), and the vascular distance further explain the significance of the location of the nodule in our model.

Cardiomegaly was also significant in our model but had negligible effect size. One possible explanation is the increased pressure in the left atria with retrograde capillary recruitment around the PN [20]. The potential influence of sex is also difficult to explain and has a negligible effect size but may be related to a higher vessel density in women than in men, as suggested in a recent study [21].

The volume measurement is unsurprisingly strongly related to the average diameter, as both relate to a different aspect (i.e., volume and diameter) of the same characteristic (i.e., size). Likewise, the volume is also related to the appropriateness of the nodule segmentation with a moderate effect size. In clinical practice, cases considered inadequate will tend to overestimate the volume (e.g., the inclusion of the bronchial wall in segmentation) and should be manually corrected. By including this variable in the model, we can minimize inter-observer variability by avoiding manual correction of the semiautomatic segmentation results but still be able to distinguish its effect from the effect attributable to cardiopulmonary hemodynamic factors.

As expected, the inter-observer agreement is very high globally but even higher in diastole, which could be related to minimizing cardiac motion using ECG-gating, as suggested by Boll et al. [18].

No reasonable inter-software agreement can be assumed, which exposes the differences in segmentation performance across different software packages, and reinforces the current recommendations that the same software package should be used throughout the follow-up period [22].

CCTA scans allow multiple independent measurements of a PN (at different phases of the cardiac cycle) in a single acquisition, controlling for most other factors (such as the absence of true growth). In this way, it is similar to the coffee-break study design, which also assumes a lack of true growth (zero-change datasets). However, the latter is more useful in phantom studies because of the increased radiation exposure [23]. Nevertheless, investigating the effect of these hemodynamic changes on PN volumetry would not be feasible with a coffee-break study design using non-gated scans (zero-change even for hemodynamic changes) and would be very inefficient in a longitudinal study (because we cannot assume the absence of true growth over time and the true volume of a PN is unknown).

We propose future research into the variability of the volume measurement in CCTA scans since growth estimation is the most critical application of volumetry in PN between 5 and 8 mm.

A limitation of the present study is the low percentage of cases included in the study from the large study sample, which is due to the large number of CCTA scans with missing systolic or diastolic phases in PACS. This is related to the department approach of sending only the most diagnostically relevant phases for archiving. Nevertheless, to our knowledge, this study represents the largest published series on PN volumetry in ECG-gated CT scans and using current and updated volumetry tools in clinical use. Another limitation of the study is the lack of information on risk factors such as smoking. Given the substantial overlap between lung cancer and CAD risk factors, we assume that their prevalence is high in the study population. We realize that the model simplifies the complex mechanisms of cardiopulmonary hemodynamics and does not attempt to address the systemic bronchial arteries or the regulatory mechanisms of ventilation and perfusion. Other non-hemodynamic factors may also influence the measurement, like the distribution of the nodules along the airways, which may also be confounded with the vascular distance.

## Conclusion

Our data show that the studied factors related to cardiopulmonary hemodynamics influence the results of volumetry tools applied to PN between 5 and 8 mm. Because the cardiac cycle phase is not statistically significant in our model, there is no optimal phase that could control this effect, but more importantly, this raises the possibility of the effect also being relevant to non-gated CT scans.

The vascular distance between the MPA and the PN and the diameter difference of the MPA between systole and diastole are related to cardiac function and resistance in the pulmonary circulation. Therefore, these factors may change between the baseline and the follow-up evaluations (e.g., decompensated heart failure) and affect the growth estimation of a PN.

The inter-observer reliability of the volumetry tools is very high, which is the motivation for automatic and semiautomatic volumetry software packages.

The study design based on ECG-gated scans seems suited to study the impact of momentary changes in the cardiopulmonary circulation on PN volumetry and is likely a good model for the potentially more extreme hemodynamic changes that a patient can experience between scans.

## Data Availability

The datasets used and analyzed during the current study are available from the corresponding author on reasonable request.

## Abbreviations

ACR: American College of Radiology
BTS: British Thoracic Society
CAD: Coronary artery disease
CCTA: Coronary CT angiography
ECG: Electrocardiogram
FOV: Field of view
I-ELCAP: International Early Lung Cancer Action Program
LD-CT: Low-dose CT scan
NELSON: Dutch-Belgian Lung Cancer Screening trial
NCCN: National Comprehensive Cancer Network
MPA: Main pulmonary artery
PACS: Picture archiving and communication system
PN: Pulmonary nodule
VDT: Volume doubling time
